# Enhanced Carbon Nanotube Ionogels for High-Performance Wireless Strain Sensing

**DOI:** 10.3390/polym17060817

**Published:** 2025-03-20

**Authors:** Xiao Wang, Menglin Tian, Jiajia Wan, Shuxing Mei, Mingwang Pan, Zhicheng Pan

**Affiliations:** 1 Department of Polymer Materials and Engineering, School of Chemical Engineering and Technology, Hebei University of Technology, Tianjin 300401, China; wangxky@163.com (X.W.); tml20001230@163.com (M.T.); wjj2944@163.com (J.W.); mwpan@126.com (M.P.); 2 Hebei Key Laboratory of Functional Polymers, Hebei University of Technology, Tianjin 300401, China; 3 State Key Laboratory of Heavy Oil Processing at Karamay, China University of Petroleum-Beijing at Karamay, Karamay 834000, China

**Keywords:** CNTs, ionogels, rigid-flexible properties, high conductivity, sensor

## Abstract

Ionogels, as emerging stretchable conductor materials, have garnered significant attention for their potential applications in flexible electronics, particularly in wearable strain sensors. However, a persistent challenge in optimizing ionogels lies in achieving a balance between enhanced mechanical properties and electrical conductivity. In this study, we successfully addressed this challenge by incorporating carbon nanotubes (CNTs) into ionogels, achieving a simultaneous improvement in the electrical conductivity (2.67 mS/cm) and mechanical properties (400.83 kPa). The CNTs served dual purposes, acting as a continuous conductive pathway to facilitate electrical signal transmission and as reinforcing nanotubes to bolster the mechanical robustness of the ionogels. Additionally, the polymer network, composed of acrylic acid (AA) and 2-hydroxyethyl acrylate (HEA), established a purely physical cross-linking network characterized by dense hydrogen bonding, which ensured sufficient toughness within the ionogels. Notably, the assembled ionogels, when utilized as wireless strain sensors, demonstrated exceptional sensitivity in detecting subtle finger movements, with the CNTs significantly amplifying the electrical response. This work provides new insights into the integration of carbon nanotubes in ionogels, expanding their applications and pioneering a fresh approach to functionalized ionogel design.

## 1. Introduction

Ionogels, formed by the uniform dispersion of ionic liquids in polymer networks [1,2,3,4], are regarded as highly promising stretchable ionic conductor materials due to their excellent ionic conductivity [5,6,7,8], thermal stability [9,10,11,12,13], mechanical properties [14,15,16,17], and flexible processability [18,19,20,21,22]. In recent years, considerable advancements have been made in the field of flexible electronics technology, with particular emphasis on the potential applications of ionogels in electronic devices [23,24,25,26] including flexible wearable strain sensors [27,28,29]. Significant progress has been achieved in the investigation of the electrical properties of ionogels, with a particular focus on enhancing their electrical conductivity and mechanical properties [30,31]. However, existing research has identified a notable trade-off between these two properties, as their simultaneous optimization remains challenging [32,33,34]. This limitation primarily arises from the role of ionic liquids, which enhance electrical conductivity with an increase their concentration but simultaneously exert an adverse impact on the mechanical performance of ionogels [35,36,37].

The electrical signal response characteristics of ionogels are primarily attributed to the localized movements of anions and cations within the ionic liquids, a process that notably enhances their conductivity [38]. However, such electrical properties are inherently constrained by their formation environment, particularly the spatial site resistances of the anions and cations that limit the movement of the ions and impede charge carrier mobility. Additionally, the impact of polymer networks on the electrical properties of ionogels cannot be overlooked. While polymer networks are essential for maintaining the overall mechanical integrity of the ionogels, excessively dense polymer networks restrict the mobility of the ionic liquids, thereby negatively impacting their electrical characteristics [39,40]. Consequently, the challenge of enhancing the mechanical properties of ionogels without compromising, and potentially even improving, their electrical performance has emerged as a critical area of ongoing research that requires further exploration.

In this context, carbon nanotubes (CNTs) have emerged as outstanding additives for simultaneously enhancing both the electrical and mechanical properties of ionogels [41,42,43,44,45] due to their remarkable electrical conductivity [46,47], distinctive structural attributes [48], and exceptionally high specific surface area [49]. The incorporation of CNTs into ionogel matrices offers dual benefits. From an electrical perspective, CNTs serve as efficient long conductive pathways. The natural electrical conductivity of CNTs, which is unaffected by the environment, also allows for the transmission of changes in electrical signals generated by the movement of anions and cations in ionic liquids [50,51]. This unique capability not only boosts the overall conductivity of the ionogels, but also enhances their sensitivity to electrical signals, facilitating the effective amplification, detection, and transmission of such signals. In terms of mechanical properties, the rigid nanotube structure of CNTs facilitates the formation of intricate, interpenetrating networks with the polymer chains within the ionogel matrix, thereby augmenting its mechanical strength and resilience. Thus, the incorporation of CNTs improve both the electrical properties and the mechanical strength of the ionogels.

To achieve the optimal mechanical properties in ionogels, the key to attaining a ’rigid-flexible’ balance lies in the effective fusion of the inherent rigidity of the CNTs and flexibility of the polymer matrices. The core value of the polymer network in ionogels is its mechanical dissipation capability, facilitating efficient stress absorption and dispersion, which is essential for the improvement in the fatigue resistance. Meanwhile, the combination of CNTs as reinforcing phases, coupled with their uniform dispersion within the ductile polymer matrix, ensures that the composites maintain their structural integrity under stress while exhibiting good deformation adaptability. The purely physical cross-linking is optimized due to the network structure formed by non-covalent interactions such as hydrogen bonding, which effectively mitigates the defects and stress concentrations introduced by chemical cross-linking, resulting in more efficient mechanical dissipation during loading and improving the overall toughness. Acrylic acid and its derivatives are particularly suitable polymer monomers due to their tunable molecular structure and versatile functional groups. The structural features not only enable the construction of energy-dissipative polymer networks, but also confer exceptional solubility and processability, thereby facilitating the preparation of high-performance ionogels.

Herein, we synthesized ionogels using the photopolymerization one-pot method by employing acrylic acid (AA) and 2-hydroxyethyl acrylate (HEA) as reactive monomers dissolved in ionic liquids (1-ethyl-3-methylimidazole ethyl sulphate, EMIES) to obtain the prepolymer solution. Furthermore, the mechanical and electrical properties of the ionogels were effectively enhanced by incorporating carbon nanotubes (CNTs) into the prepolymer solution. The purely physical crosslinking network, which relies on the formation of dense hydrogen bonds as crosslinking points, plays a critical role in ensuring the toughness of the ionogel systems. CNTs not only enhance the mechanical properties of ionogels, but also establish conductive pathways, resulting in a significant improvement in mechanical strength to 400.83 kPa and electrical conductivity to 2.67 mS/cm. The CNTs form conductive pathways that effectively detect minute movements within the ionic liquids, facilitating a highly sensitive electrical signal response. Utilizing this capability, a wireless physiological signal monitoring system was developed, capable of accurately detecting physiological signals both remotely and wirelessly. The rigid-flexible structural design of this system effectively integrates the electrical properties of the ionogel, positioning it as a promising material for applications in flexible electronic devices.

## 2. Materials and Methods

### 2.1. Materials

Acrylic acid (AA, >99%) was purchased from Shanghai Macklin Biochemical Technology Co., Ltd. (Shanghai, China). 2-Hydroxyethyl acrylate (HEA, 95.8%), 1-ethyl-3-methylimidazolium ethylsulfate (EMIES, 99.99%), photoinitiator 1173 (2-hydroxy-2-methylpropiophenone, 99.29%), and multi-walled carbon nanotubes (CNTs) (inter diameter: 5–12 nm, outer diameter: 30–50 nm, length: 10–20 μm) were purchased from Bide Pharmatech Ltd. (Shanghai, China). All reagents were not further purified.

### 2.2. Preparation of PHA Ionogels

AA and HEA were added to EMIES and sonicated for 0.5 h to mix well and form a prepolymer solution. Photoinitiator 1173 was added to the prepolymer solution and mixed well by ultrasonication for 0.5 h. After venting N_2_ for 30 min, PHA ionogels were synthesized by UV irradiation (365 nm, 72 W) for 1 h. The reaction ratio reference is shown in Table S1.

### 2.3. Preparation of PHAC Ionogels

CNTs were added to the prepolymer solution formed by AA, HEA, and EMIES and sonicated for 1 h for uniform dispersion. After that, photoinitiator 1173 was added and sonicated for 0.5 h to dissolve it uniformly. Finally, after venting N_2_ for 30 min, UV irradiation (365 nm, 72 W) for 1 h was used to obtain PHAC ionogels. The reaction ratio reference is shown in Table S2.

### 2.4. Characterizations and Measurements

FTIR spectroscopy (Bruker Tensor-27 spectrometer, Ettlingen, Germany) was used to determine the chemical structures of the PHA ionogels and PHAC ionogels. Observation of the ionogels’ cross-section morphologies was carried out through field-emitting scanning electron microscopy (FESEM) using a (Thermo APreo 2S instrument Waltham, MA, USA). Gold sputter-coating preceded the imaging process. Field-emission scanning electron microscopy (Thermo APreo 2S, Waltham, MA, USA) was used for EDS mapping of the PHAC ionogels to determine the elemental distribution. Rheology-related tests were performed by means of a rotational rheometer (MCR 302e, from Austria). Temperature-variable FTIR spectra were collected in transmission mode on an FTIR (Bruker INVENIO-S, German) spectrometer from 20 to 130 °C with an interval of 25 °C. All of the temperature-dependent FTIR spectra from 20 to 130 °C were used to perform 2DCOS analysis. 2DCOS analysis was carried out using the two-dimensional correlation spectroscopy software (2DCS Professional I-002-great-1.4B), 2DCS professional version (State Key Laboratory of Polymer Materials Engineering, Sichuan University). In the contour maps, red colors are defined as positive intensities, while blue colors are negative ones.

### 2.5. Mechanical Properties Measurements

The mechanical properties of the PHA ionogels and PHAC ionogels were quantified utilizing a CMT6104 universal testing machine (XinSanSi, Shenzhen, China) with tensile stress–strain variations at a tensile rate of 100 mm min^−1^. The sample was a rectangle 40 mm long, 5 mm wide and 2 mm thick. The tensile toughness (τ) can be calculated by integrating the area under the stress (σ)–strain (ε) curve. The equation used is given below:

(1) $$\tau =\int_{0}^{\varepsilon_{\max}}\sigma d\varepsilon$$

where σ is the engineering stress of the sample, ε is the engineering strain, and ε_max_ is the elongation at break.

### 2.6. Adhesion Properties

The adhesion properties of the ionogels were tested at room temperature using the lap-shear method and a universal testing machine (CMT6104). The ionogels were bonded between the substrates, pressed with a 100 g weight at 60 °C for 12 h, and then stretched at 100 mm/min until the ionogels and substrates were completely separated. Three experiments were performed for each sample. The adhesion strength was calculated using the following equation:

Adhesion Strength = F*max*/S (2)

where F*max* (N) represents the maximum force during stretching, and S (m^2^) represents the adhesion area.

For repeated adhesion experiments, lap-shear experiments were performed using the same method described above.

### 2.7. Electrochemical Testing of PHA Ionogels and PHAC Ionogels

Electrochemical impedance measurements were performed on the PAS-E ionogels using an electrochemical workstation (CHI760E). All samples were subjected to this experiment. The conductivity σ (mS/cm) of the ionogels was calculated using the following equation:

σ = L/(SR) * 1000 (3)

where L (cm), R (Ω), and S (cm^2^) are the distance between the two electrodes, the resistance of the ionogel, and the area of the electrode sheet, respectively.

In addition, the resistance change signal of the ionogel was investigated by coupling the electrochemical workstation (CHI760E) to a universal testing machine (CMT6104). The rate of resistance change of the ionogel (ΔR/R_0_) and the specification factor GF were calculated using the following formula:

ΔR/R_0_ = (R − R_0_)/R_0_ (4)

where R (Ω) and R_0_ (Ω) are the resistance of the ionogel after the application of strain and the initial resistance of the ionogel, respectively.

GF = (ΔR/R_0_)/ε (5)

where ε (%) is the strain of the ionogel.

The ionogel sensing test was prepared by sandwiching a 40 mm × 5 mm × 2 mm rectangular ionogel on commercial VHB tape to form a flexible ionogel sensor with copper electrodes and wires attached to both ends of the ionogel. The sensors were then attached to the skin of volunteers to monitor the electrical signals generated during exercise. The above experiments were carried out using an electrochemical workstation (CHI760E) to record the electrical signals.

The wireless signal transmission kit was purchased from Shenzhen Runeskee Technology Co., Shenzhen, China. The sensor kit includes a signal converter, a wireless signal exporter and a wireless signal receiver, and power supply kit. The PHAC ionogel electrode is assembled by connecting a signal transmitter through the electrode leads in each of the two parts of the ionogel and then calibrating the ionogel against the pain over weights.

Informed written consent was obtained from all human participants prior to the research.

## 3. Results and Discussion

### 3.1. Formation of the Ionogels

In this rigid-flexible structure design, the polymer network is required to provide enhanced mechanical dissipation to ensure the toughness of the ionogel by establishing a physically cross-linked system rather than a chemically cross-linked one. Specifically, acrylic acid (AA), 2-hydroxyethyl acrylate (HEA), and ionic liquids (1-ethyl-3methyl ethyl sulfate, EMIES) were selected as the primary components. The PH*x*A*y* ionogels were synthesized by mixing AA and HEA in EMIES to form a prepolymer solution, followed by the addition of photoinitiator 1173 to facilitate in situ polymerization under UV irradiation. Here, the molar ratio of HEA (denoted as *x*) to AA (denoted as *y*) in the monomers was systematically varied (0:1, 1:2, 1:1, 2:1, and 1:0, respectively) while maintaining a constant ratio of the total molar amount of AA and HEA to the molar amount of EMIES at 3:2 (Table S1).

The scalability of CNT composite ionogel manufacturing depends on optimizing material integration and process adaptability for industrial frameworks. Key approaches include solvent-free UV-curing or extrusion-based 3D printing, which align with roll-to-roll coating or injection molding for large-scale production, provided that uniform CNT dispersion (via high-shear mixing or surface modification) and ionic liquid compatibility are ensured. Challenges such as CNT agglomeration in bulk processing, long-term mechanical stability under stress, and the high cost of ionic liquids must be addressed. However, their compatibility with flexible substrates (e.g., textiles, plastics) and the use of energy-efficient curing methods position them as promising candidates for industrial applications in wearables, solid-state batteries, or printed sensors. Collaborative efforts to standardize formulations and reduce the costs of raw material could accelerate the transition from lab-scale innovation to commercial viability.

**Figure 1:**
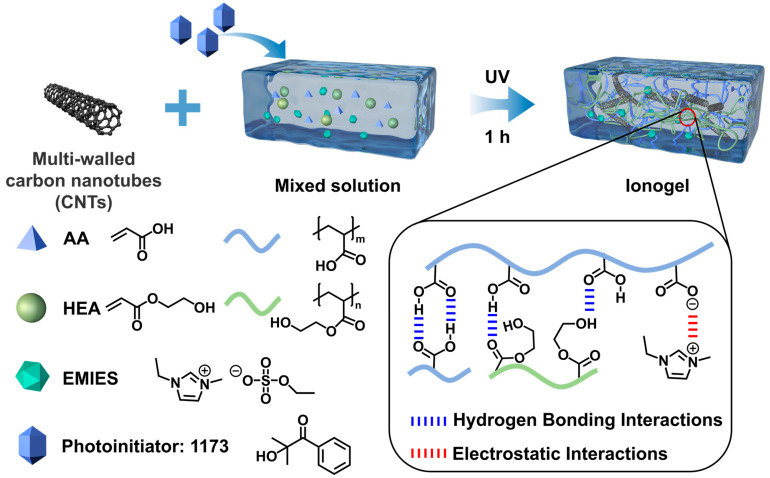
Synthesis methods and design concepts for IMPS ionogels. The monomers AA and HEA were first homogeneously dissolved in the ionic liquid EMIES to form a prepolymer solution, and then the CNTs were homogeneously dispersed in the prepolymer solution. Finally, photoinitiator 1173 was added and the ionogels were formed by UV irradiation in one step. In the ionogels, AA and HEA can form many different hydrogen bonds, and this dense hydrogen bonding ensures the stability of the ionogel. AA can also form electrostatic interactions with EMIES, which ensures the stability of EMIES in the ionogel without loss.

The PH_1_A_2_ ionogel was selected for further investigation due to its optimized mechanical and adhesion properties. PHAC*m* ionogels were then prepared by dispersing carbon nanotubes (CNTs) in the prepolymer solution, where *m* wt% represents the mass ratio of CNTs to the prepolymer solution (Table S2). As illustrated in Figure 1, the ionogel exhibited a diverse array of physical interactions, including extensive hydrogen bonding and electrostatic interactions among the monomers and ILs, which collectively form dense physical networks that are crucial for ensuring the stable formation of the ionogel. Hydrogen bonding interactions that formed between HEA and EMIES as well as electrostatic interactions and hydrogen bonding interactions formed between AA and EMIES played a key role in stabilizing the ionic liquids in the polymer network to prevent them from leaching (Figure S1). The entanglement of polymer molecular chains with CNTs further ensures the robust incorporation of CNTs within the ionogels. CNTs and polymer chains cooperate to form ionogels with both mechanical strength and toughness, retaining the properties of ionic liquids while enhancing the electrical conductivity, achieving a multifaceted performance enhancement of the ionogels.

### 3.2. Structure and Morphology of the Ionogels

**Figure 2:**
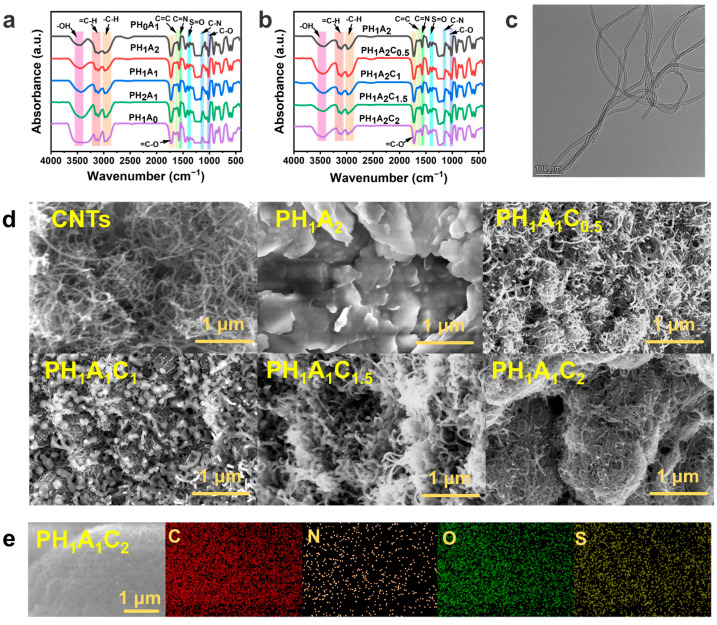
Characterization of the morphology, structure, and elemental distribution of the ionogels. (**a**) FTIR spectra of PH_0_A_1_, PH_1_A_2_, PH_1_A_1_, PH_2_A_1_, and PH_1_A_0_. (**b**) FTIR spectra of PH_1_A_2_, PH_1_A_2_C_0.5_, PH_1_A_2_C_1_, PH_1_A_2_C_1.5_, and PH_1_A_2_C_2_. (**c**) TEM images of CNTs. (**d**) Cross-sectional SEM images of PH_1_A_2_, PH_1_A_2_C_0.5_, PH_1_A_2_C_1_, PH_1_A_2_C_1.5_, and PH_1_A_2_C_2_. (**e**) Elemental mapping images of PH_1_A_2_C_2_ (C, N, O, and S).

The structure of the ionogels was characterized by Fourier transform infrared spectroscopy (FTIR) (Figure 2a). The characteristic peak at 3434 cm^−1^ was attributed to hydroxyl (O-H) stretching vibrations arising in the side groups of the polymer chains, originating from the carboxyl group of AA and the hydroxyl group of HEA. The infrared absorption peaks at 3156 cm^−1^ and 3106 cm^−1^ were attributed to C-H stretching vibrations of the unsaturated hydrocarbon groups, originating from the imidazole in EMIES. The characteristic peak at 2983 cm^−1^ was the C-H stretching vibration on the saturated hydrocarbon group, while the characteristic peak at 1724 cm^−1^ was attributed to the carbonyl (C=O) stretching vibration, originating from the AA and HEA. The IR spectra at 1571 cm^−1^ and 1168 cm^−1^ showed the characteristic stretching vibration of imidazole, which was attributed to the C=N and C-N in the EMIES, respectively. In addition, the absorption peaks in the IR spectra at 1394 cm^−1^ and 1016 cm^−1^ were attributed to the asymmetric stretching vibration of S=O and the stretching vibration of C-O of the ethyl sulfate group in EMIES, respectively. The above results indicate that the PHA ionogels had been successfully synthesized.

Similarly, the presence of identical infrared absorption vibrational peaks in the PHAC ionogels confirmed the successful synthesis of our PHAC ionogels (Figure 2b). Furthermore, the incorporation of CNTs into the ionogels resulted in a transformation of the PHAC ionogels into a pure black and opaque material, in stark contrast to the transparent nature of the PHA ionogels (Figure S2). Transmission electron microscopy (TEM) images revealed that the CNTs possessed a long, fibrous, hollow tubular morphology, aligning perfectly with our design intentions (Figure 2c). Scanning electron microscopy (SEM) provided additional insights into the morphology of the CNTs and the cross-sectional structure of the ionogels (Figure 2d), showing that the CNTs were typically dispersed as fluffy nanotubes. In the PH_1_A_2_ ionogel, a multilayered porous structure was apparent, demonstrating that the ionic liquids were afforded ample space for effective distribution. In the PHAC ionogels, efficient aggregation of CNTs and polymers was observed, with a regular increase in range as the CNT content increased. The elemental distribution in the PH_1_A_2_C_2_ ionogels was examined using energy dispersive spectroscopy (EDS). The results revealed a uniform distribution of carbon (C), nitrogen (N), oxygen (O), and sulfur (S) elements throughout the ionogel, suggesting that the ionogels were homogeneously synthesized (Figure 2e and Table S3).

Rotational rheometry was used to characterize the rheology of the ionogels. The viscosity of the prepolymer solution increased with increasing CNT content, which was attributed to the fact that the higher aspect ratio of the CNTs formed a three-dimensional osmotic network during dispersion, thereby increasing the flow resistance of the ionic liquids (Figure S3). In addition, π–π interactions between the CNTs and ionic liquids contributed to the increased viscosity of the system while enhancing the energy storage modulus of the ionogels. However, the increase in viscosity may hinder the diffusion and polymerization reaction of the monomers. This adverse effect can be mitigated by employing a brief period of rapid UV photopolymerization.

**Figure 3:**
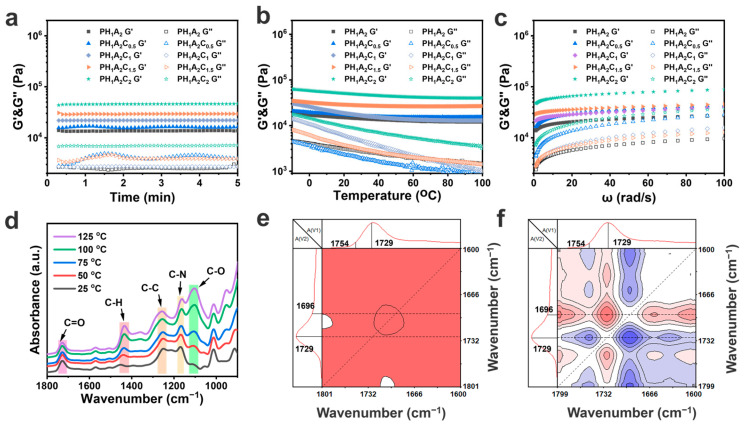
Rheology and 1D/2D infrared of the ionogels. (**a**) Stability of the energy storage modulus (G′) and loss modulus (G″) of PH_1_A_2_, PH_1_A_2_C_0.5_, PH_1_A_2_C_1_, PH_1_A_2_C_1.5_, and PH_1_A_2_C_2_ with time. (**b**) Variation in the energy storage modulus (G′) and loss modulus (G″) of PH_1_A_2_, PH_1_A_2_C_0.5_, PH_1_A_2_C_1_, PH_1_A_2_C_1.5_, and PH_1_A_2_C_2_ with temperature. (**c**) Variation in the energy storage modulus (G′) and loss modulus (G″) of PH_1_A_2_, PH_1_A_2_C_0.5_, PH_1_A_2_C_1_, PH_1_A_2_C_1.5_, and PH_1_A_2_C_2_ with frequency. (**d**) Infrared intensity changes curves of P H_1_A_2_C_2_ for 1800 cm^−1^–900 cm^−1^ at 25 °C, 50 °C, 75 °C, 100 °C, and 125 °C. (**e**) Synchronized spectra of 2DCOS generated by (**d**). Red represents positive intensity and blue represents negative intensity. (**f**) Asynchronized spectra of 2DCOS generated by (**d**). Red represents the positive intensity and blue represents the negative intensity.

The energy storage modulus (G′) of the PHAC ionogels gradually increased with the increase in CNT content, and both were higher than that of the PH_1_A_2_ ionogels (Figure 3a). With the increase in temperature, the energy storage modulus (G′) and loss modulus (G″) of the ionogels exhibited a certain degree of stability, although a gradual decline was observed (Figure 3b). This behavior can be attributed to the gradual dissociation of the physical cross-linking points at increased temperatures, coupled with the progressive unraveling of the entangled polymer molecular chains. The energy storage modulus (G′) was always greater than the loss modulus (G″), indicating that despite the gradual dissociation of the non-covalent bonds, the physical cross-linked network retained a certain degree of structural integrity, thereby maintaining dominant elastic behavior.

In the range of 1 rad/s to 100 rad/s, the energy storage modulus (G′) and loss modulus (G″) of the ionogel exhibited minimal variation, with the G′ consistently exceeding the G″, indicating the structural stability of the ionogel (Figure 3c). This suggests that the network structure remained intact over the frequency range tested and that the energy was mainly stored in the form of an elastic potential rather than being dissipated by viscous flow. The increase in energy storage modulus (G′) with an increase in the CNT content could be attributed to the gradual expansion of the aggregation region between the CNTs and polymer network, which facilitated the formation of a larger homogeneous aggregate. This structural reconfiguration constrains polymer chain movement, which is consistent with the cross-sectional morphology observed in the SEM images (Figure 2d).

Variable temperature Fourier transform infrared (VT-FTIR) was used to characterize the fine structural changes within the ionogels (Figure 3d and Figure S4). The C=O stretching vibration peak (1727 cm^−1^) originating from the polymer molecular chain (AA and HEA), the C-H in-plane bending vibration (1434 cm^−1^), and the C-C stretching vibration (1255 cm^−1^) associated with both the polymer molecular chain and the imidazole moiety in the EMIES exhibited a significant enhancement with increasing temperature. The O-H stretching vibrations (3650 cm^−1^) originating from AA and HEA also significantly increased the intensity of the absorption peak (Figure S5). The same regular enhancement was observed for the C-N stretching vibration (1162 cm^−1^) originating from the imidazole and the C-O stretching vibration (1103 cm^−1^) on the ethyl sulfate moiety in EMIES. These results indicate that upon heating, the activity of the groups within the PH_1_A_2_C_2_ ionogel is enhanced, leading to the dissociation of the physical cross-linking points, including hydrogen bonding interactions and electrostatic interactions, which is consistent with the previous rheological results.

While temperature-induced weakening of hydrogen bonding is a well-known phenomenon, the hierarchical dissociation sequence of multiple hydrogen bonding interactions in this system requires more detailed mechanistic analysis. Two-dimensional correlation spectra (2DCOS) were generated from all temperature-dependent IR spectra to extract more relevant details (Figure 3e,f and Table S4). According to Noda’s judgement rule based on the sign of the cross peaks in the synchronous and asynchronous spectra, the order of the C=O correlated species on heating was determined as: 1729 → 1754 → 1696 (→denotes prior or earlier than; see Table S3 for details of the determination in the Supplentary Materials) [52,53]. The three peaks above may correspond to the free C=O_free_ (1754 cm^−1^) that is not hydrogen bonded, the disordered C=O_disordered_ (1729 cm^−1^) that is hydrogen bonded, and the ordered C=O_ordered_ (1696 cm^−1^) that is hydrogen bonded [54,55]. This suggests that on heating, the disordered C=O dissociates preferentially to form free C=O, while the ordered C=O_ordered_ (including the hydrogen bonding of cyclic dimers formed in AA) dissociates at a later stage. This also explains the excellent temperature stability of ionogels.

### 3.3. Mechanical Properties of the Ionogels

**Figure 4:**
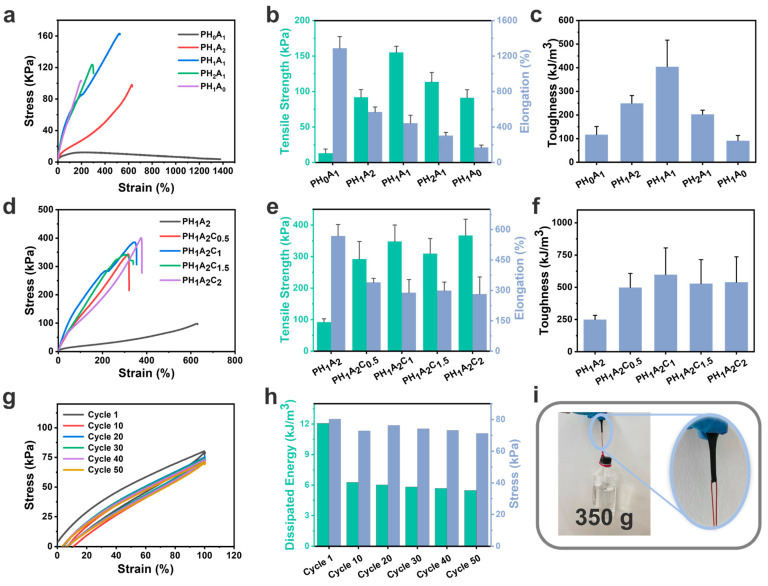
Mechanical properties of the PHA and PHAC ionogels. (**a**) Stress–strain curves of PH_0_A_1_, PH_1_A_2_, PH_1_A_1_, PH_2_A_1_, and PH_1_A_0_. (**b**) Tensile strength and elongation obtained from the stress–strain curves of PH_0_A_1_, PH_1_A_2_, PH_1_A_1_, PH_2_A_1_, and PH_1_A_0_. (**c**) Toughness calculated by the integration of PH_0_A_1_, PH_1_A_2_, PH_1_A_1_, PH_2_A_1_, and PH_1_A_0_ from the stress–strain curves. (**d**) Stress–strain curves of PH_1_A_2_, PH_1_A_2_C_0.5_, PH_1_A_2_C_1_, PH_1_A_2_C_1.5_, and PH_1_A_2_C_2_. (**e**) Tensile strength and elongation obtained from the stress–strain curves of PH_1_A_2_, PH_1_A_2_C_0.5_, PH_1_A_2_C_1_, PH_1_A_2_C_1.5_, and PH_1_A_2_C_2_. (**f**) Toughness calculated by the integration of PH_1_A_2_, PH_1_A_2_C_0.5_, PH_1_A_2_C_1_, PH_1_A_2_C_1.5_, and PH_1_A_2_C_2_ from the stress-strain curves. (**g**) Stretching curves for 50 cycles of the PH_1_A_2_C_2_ ionogels. (**h**) Toughness and maximum stress obtained by stretching curves for 50 cycles of the PH_1_A_2_C_2_ ionogels. (**i**) Images of the PH_1_A_2_C_2_ cross-section that remained unbent after hanging a 350 g weight.

A mechanical universal testing machine was used to characterize the mechanical properties of the PHA ionogels and PHAC ionogels (Figure 4). For the PHA ionogels, the strength of the ionogels increased significantly with the increase in the HEA content, from 12.28 kPa of PH_0_A_1_ up to 163.13 kPa of PH_1_A_1_ (Figure 4a). This was due to the fact that the HEA monomers had more hydrogen bonding supply sites relative to the AA monomers, which allowed for the formation of more and denser hydrogen bonding networks, enhancing the interaction forces between molecular chains (Figure 4b). The mechanical strength of PH_1_A_0_ was much higher than that of PH_0_A_1_, proving that the strength and stability of the hydrogen bonding network structure formed between HEA and EMIES was higher than that of the physical cross-linking structure that formed between AA and EMIES. The toughness values calculated from the stress–strain curves showed the same regularity (Figure 4c). However, when the HEA content was further increased, both the strength and toughness decreased. This is because the overly dense hydrogen bonding networks restricted the movement of the polymer chains, preventing the material from efficiently absorbing and dissipating energy through the movement of the chain segments under the external stress. Additionally, the dense networks prevented the rearrangement and combination of the chain segments during the repair process of the physical cross-links. As a result, the overly dense cross-linking points led to a slight reduction in tensile strength with the excessive addition of HEA.

With the addition of CNTs alone, the strength of the ionogel increased again and could reach up to 400.83 kPa of PH_1_A_2_C_2_ (Figure 4d). This proves that CNTs effectively serve as a rigid and stiff network structure to improve the mechanical properties of ionogels. While the incorporation of CNTs initially led to a significant improvement in the tensile strength of the ionogel, subsequent changes in the CNT content had a limited impact on the ionogel’s strength (Figure 4e). Meanwhile, the toughness of the ionogels increased with the addition of CNTs due to interwoven networks allowing for energy dissipation; however, no significant changes were observed with variations in the CNT content (Figure 4f). The PH_1_A_2_C_2_ was tested by 50 cyclic tensile tests in the 100% strain range, revealing a reduction in hysteresis with an increasing number of tensile cycles, while the strength remained stable, demonstrating excellent cyclic stability and fatigue resistance (Figure 4g,h). These phenomena can be attributed to the rapid reorganization of physical cross-linking points within the ionogel. In addition, a 5 mm wide and 2 mm thick sample of the PH_1_A_2_C_2_ ionogel could easily lift a 350 g weight without sustaining any damage (Figure 4i).

### 3.4. Adhesion Properties of the Ionogels

**Figure 5:**
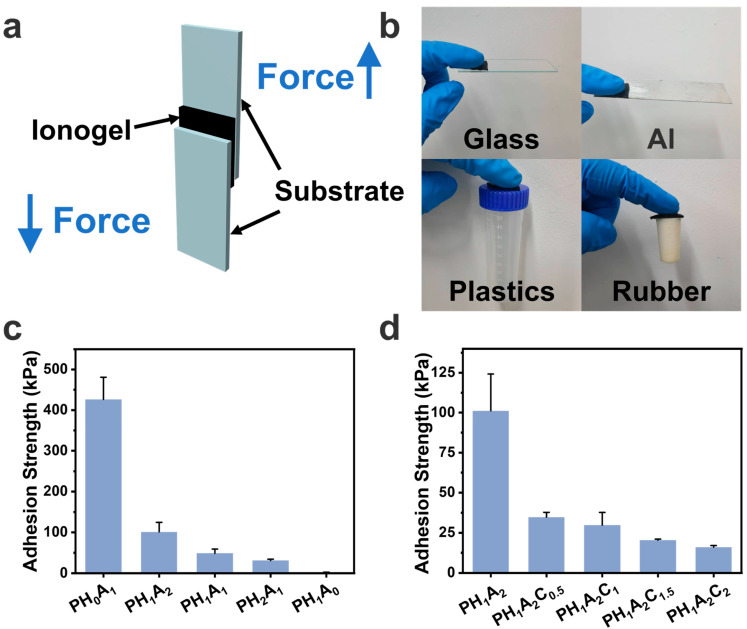
Adhesive properties of the PHA ionogels and PHAC ionogels. (**a**) Lap shear schematic. (**b**) Images of PH_1_A_2_C_2_ ionogels adhering to different substrates including glass, Al sheets, plastic, and rubber. (**c**) Adhesion strength of PH_0_A_1_, PH_1_A_2_, PH_1_A_1_, PH_2_A_1_, and PH_1_A_0_ ionogels. (**d**) Adhesion strength of PH_1_A_2_, PH_1_A_2_C_0.5_, PH_1_A_2_C_1_, PH_1_A_2_C_1.5_, and PH_1_A_2_C_2_ ionogels.

The prepared ionogels, which were capable of wetting the substrate surface, formed extensive interfacial interactions, such as hydrogen bonding and electrostatic interactions, conferring strong adhesion properties, as confirmed by subsequent lap-shear experiments (Figure 5a). The PH_1_A_2_C_2_ ionogels demonstrated the ability to adhere to various substrate surfaces, such as glass, Al sheets, plastics, and rubber, thereby broadening their potential application scenarios (Figure 5b). As shown in Figure 5c, the adhesion strength of the PHA ionogel gradually decreased with a decrease in the AA content by performing lap-shear experiments on Al sheets. This is because, as the Young′s modulus of the ionogel increased, the ionogel could not wet the substrate surface sufficiently. Additionally, the decreased AA content also hindered the formation of sufficient electrostatic interactions between the ionogel and the substrate (Figure S6). The selection of the PH_1_A_2_ ionogel for the subsequent phase of the investigation was primarily driven by its favorable mechanical properties and strong adhesion characteristics. Similarly, the adhesion of the PHAC ionogel decreased with increasing CNT content, which was attributed to the substantial increase in the Young′s modulus of the ionogel with the addition of CNTs; simultaneously, the CNTs slightly disrupted the ionogel-to-substrate interaction (Figure 5d and Figure S7).

### 3.5. Electrical Properties of Ionogels and Wireless Physiological Signal Sensors

**Figure 6:**
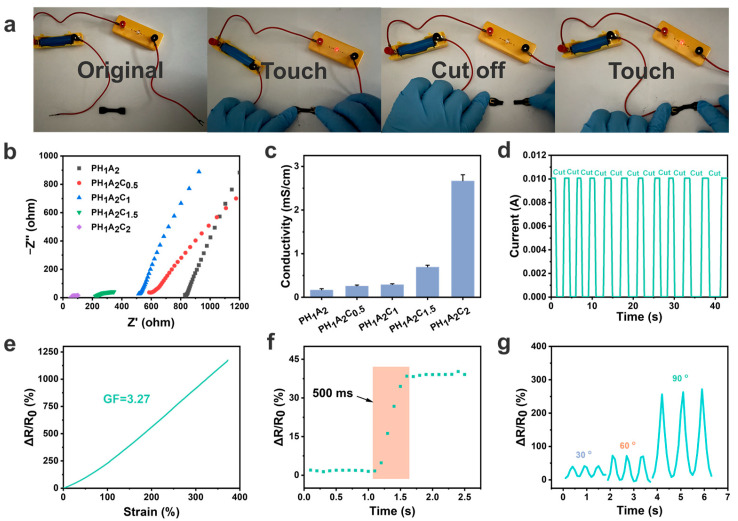
Electrical application characteristics for the PHA and PHAC ionogels. (**a**) PH_1_A_2_C_2_, as a wire to connect the current path, was cut off when the small bulb was off and reconnected, the small bulb was bright. (**b**) Nyquist plots of PH_1_A_2_, PH_1_A_2_C_0.5_, PH_1_A_2_C_1_, PH_1_A_2_C_1.5_, and PH_1_A_2_C_2_ measured by the EIS impedance method. (**c**) Conductivity of PH_1_A_2_, PH_1_A_2_C_0.5_, PH_1_A_2_C_1_, PH_1_A_2_C_1.5_, and PH_1_A_2_C_2_ calculated from the Nyquist plots. (**d**) PH_1_A_2_C_2_ current plot for 10 cycles of contact after cut off. (**e**) PH_1_A_2_C_2_ response time to strain. (**f**) GF of PH_1_A_2_C_2_ was calculated to strain ranges of 300%. (**g**) Current output signals of strain sensors under different deformations (30°, 60°, and 90°).

CNT composite ionogels hold promise for a variety of applications in flexible electronics and energy systems, including supercapacitor electrodes, responsive electronic skins, and thermoelectric collectors, due to their tunable electrical conductivity and mechanical properties. We assembled ionogels into flexible sensors. When subjected to certain external stimuli such as pressure changes or stretching, the density, velocity, and conductive pathways of ionic migration change, resulting in the output of different electrical signals. The electrical resistance of the ionogels was tested using the electrochemical impedance spectroscopy (EIS) method via an electrochemical workstation, which showed a significant decrease in the resistance with the increase in CNT content (Figure 6b). This phenomenon was attributed to the high conductivity of CNTs and their long fiber-like structure, which established more current pathways within the ionogel matrix. The calculated conductivity of PH_1_A_2_C_2_ exhibited its high electrical performance, aligning with the requirements for flexible sensors considering its modulus and toughness (Figure 6c). The same pattern was observed when the CNT content was increased based on the PH_1_A_1_ ionogel (Figure S8).

In the PHAC ionogel system, the impedance slope showed a characteristic evolution of ‘falling-rising-falling′, which represents the interfacial charge diffusion ability and is closely related to the reconstruction of the ion transport path regulated by the CNT content. In the initial stage, the addition of CNTs forms a three-dimensional permeation network, increasing the flow resistance of ionic liquids, which slows the charge diffusion and results in a decrease in the slope. As the CNT content increases, a continuous conductive network is formed, optimizing the ionic transport paths and accelerating the ion diffusion, leading to an increase in the slope. However, further increases in the CNT content increase the resistance to the ionic diffusion, causing the slope to decrease for a second time. A current path experiment confirmed this capacity, wherein using PH_1_A_2_C_2_ as a conductor successfully illuminated a small bulb. When PH_1_A_2_C_2_ was cut off, the current path was interrupted, causing the bulb to extinguish (Figure 6a). When the cut off PH_1_A_2_C_2_ was reconnected, the small bulb lit up again, demonstrating that the ionogel could act as an electrical self-healing conductor.

Subsequent current tests revealed that each time PH_1_A_2_C_2_ was cut and reconnected, the current quickly stabilized, maintaining consistency with the initial reading even after 10 cycles (Figure 6d). In addition, a flexible strain sensor prototype was constructed, exhibiting an electrical response time of only 500 ms when subjected to tensile strain (Figure 6e). The strain factor (GF), defined as the sensitivity of the ionogels to mechanically induced changes in electrical resistance, was calculated to be 3.27 and remained stable across a strain range of up to 350% and more (Figure 6f). The GF of this work exceeded some of the recent reports in the literature and showed that our ionogels have good strain sensitivity (Figure S9) [56,57,58,59,60,61]. This phenomenon was attributed to the movement of the ionic liquids under stress, which generated changes in current, while the long conductive paths formed by the CNTs effectively captured and stabilized these responsive electrical signals. Signal detection experiments were performed on the assembled flexible strain sensors, which demonstrated the ability to transmit electrical signals of varying strengths depending on the degree of bending with high sensitivity (Figure 6g). The ionogel sensors could monitor motion signals when stretched as well as in different shapes such as fingers and wrists (Figure S10). In particular, they could accurately detect and display stepped electrical signals when fingers were bent at different angles.

**Figure 7:**
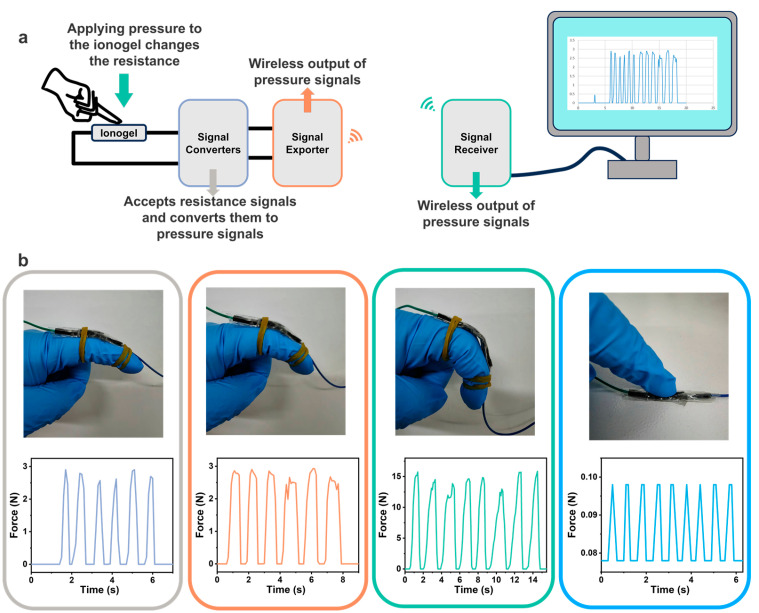
Wireless physiological signal sensor based on the PH_1_A_2_C_2_ ionogel. (**a**) Schematic diagram of the assembly mechanism of the wireless physiological signal sensor including the PH_1_A_2_C_2_ ionogel electrodes, signal converter, wireless signal exporter, wireless signal acceptor, and computer. (**b**) Capture of various gesture signals by the PH_1_A_2_C_2_ ionogel sensor (The lines on each figure correspond to the signals of the relevant action).

Physiological signal monitoring technology has garnered significant attention in recent years. We successfully developed a set of wireless signal physiological sensors based on the PH_1_A_2_C_2_ ionogel (Figure 7a and Figure S11) where the sensor adopts the PH_1_A_2_C_2_ ionogel as the sensing material instead of conventional electrodes. It converts resistance signals to pressure signals (unit: N) via a signal conversion module. Equipped with wireless transmission functionality, the sensor transmits converted signals in real-time to a signal-receiving module, allowing users to easily read and analyze the data through the computer. Wearing the sensor on the volunteer’s finger, it effectively captured the relevant movement of the finger and displayed distinct signals corresponding to different motion forms (Figure 7b). Notably, the wireless sensor exhibited high sensitivity, detecting forces as low as 0.1 N with gentle finger presses. In addition, under identical motion capture conditions, the PH_1_A_2_C_2_ ionogel showed double the signal intensity compared with the PH_1_A_2_ ionogel, validating the critical role of CNTs in amplifying electrical signals (Figure S12). This technology not only improves the convenience and accuracy of monitoring, but also presents a promising prospect for applications in medical diagnostics and health management.

## 4. Conclusions

In this paper, we successfully prepared stiff and flexible PHAC ionogels by incorporating CNTs, achieving the simultaneous enhancement of both the mechanical properties (including strength and toughness) and electrical conductivity. The tensile strength of the PHAC ionogels was enhanced up to 400.83 kPa while maintaining an excellent electrical conductivity of 2.67 mS/cm. Subsequently, the role of hydrogen bonding in the structure was demonstrated using temperature-dependent infrared spectroscopy and two-dimensional infrared, revealing the dense hydrogen bonding, and the rigid CNTs collectively contributed to the stability of the ionogels under diverse environmental conditions. We further evaluated the electrical properties of the ionogels and developed a wireless physiological signal sensor based on the ionogels. The sensor exhibited the ability to detect subtle finger movements and wirelessly transmit the relevant data to a computer. This research not only broadens the application scope of ionogel materials, but also new subtle finger insights for the development of high-performance and multi-functional flexible electronic devices.

## Data Availability

The data that support the findings of this study are available from the corresponding author upon reasonable request.

## Supplementary Materials

The following supporting information can be downloaded at: https://www.mdpi.com/article/10.3390/polym17060817/s1, Figure S1: Images of PH0A1, PH1A2, PH1A1, PH2A1, and PH1A0 after ionogel synthesis; Figure S2: Images of PH1A2C0.5, PH1A2C1, PH1A2C1.5, and PH1A2C2 after ionogel synthesis; Figure S3: The viscosity of PH1A2C0.5, PH1A2C1, PH1A2C1.5, and PH1A2C2 ionogels; Figure S4: Infrared intensity change curves of P H1A2C2 for 4000 cm^−1^–400 cm^−1^ at 25 °C, 50 °C, 75 °C, 100 °C and 125 °C; Figure S5: Infrared intensity change curves of P H1A2C2 for 4000 cm^−1^–2700 cm^−1^ at 25 °C, 50 °C, 75 °C, 100 °C and 125 °C; Figure S6: Young’s modulus of PH0A1, PH1A2, PH1A1, PH2A1, and PH1A0 ionogels; Figure S7: Young’s modulus of PH1A2C0.5, PH1A2C1, PH1A2C1.5, and PH1A2C2 ionogels; Figure S8: Electrical application characteristics for PHA and PHAC ionogels. (a) Nyquist plots of PH1A1, PH1A1C0.5, PH1A1C1, PH1A1C1.5, and PH1A1C2 measured by the EIS impedance method. (b) Conductivity of PH1A1, PH1A1C0.5, PH1A1C1, PH1A1C1.5, and PH1A1C2 calculated from Nyquist plots; Figure S9: Comparison of GF with previously reported ionogel sensors; Figure S10: Performance of the ionogel sensors. (a) Electrical response of the ionogel sensor when stretched. (b) Electrical response of the ionogel sensor when adhered to the finger. (c) Electrical response of the ionogel sensor when adhered to the wrist. (d) Ionogel sensor producing graded electrical signals depending on the degree of finger bending; Figure S11: Wireless sensor kit assembled from PH1A2C2 ionogel; Figure S12: Comparison of the sensing signals of PH1A2C2 ionogel and PH1A2 ionogel for small finger bends; Table S1: Formula dosage table of PHA ionogels; Table S2: Formula dosage table of PHAC ionogels; Table S3: EDS elemental analysis total content of PH1A2C2 ionogel; Table S4: Final results of the multiplication of the signs of each cross-peak in 2DCOS synchronous and asynchronous spectra of the ionogel (Figure 3d,e).
