# Supplementary material for: Enhanced Carbon Nanotube Ionogels for High-Performance Wireless Strain Sensing
Source: Polymers (Basel). 2025 Mar 20;17(6):817. doi: 10.3390/polym17060817 (PMC11946641; doi:10.3390/polym17060817)
Supplement: Supplementary file 1 [file polymers-17-00817-s001.zip › polymers-3512668-supplementary.pdf]

Supporting Information

Enhanced Carbon Nanotube Ionogels for High-Performance Wireless Strain Sensing

Xiao Wang [1], Menglin Tian [1], Jiajia Wan [1], Shuxing Mei [2,], Mingwang Pan [1,3] and Zhicheng Pan [1,3,*]*

1 Department of Polymer Materials and Engineering, School of Chemical Engineering and Technology, Hebei University of Technology, Tianjin 300401, China; wangxky@163.com (X.W.); tml20001230@163.com (M.T.); wjj2944@163.com (J.W.); mwpan@126.com (M.P.)

2 State Key Laboratory of Heavy Oil Processing at Karamay, China University of Petroleum-Beijing at Karamay, Karamay 834000, China

3 Hebei Key Laboratory of Functional Polymers, Hebei University of Technology, Tianjin 300401, China

* Correspondence: shuxingmei@cupk.edu.cn (S.M.); panz@hebut.edu.cn (Z.P.)

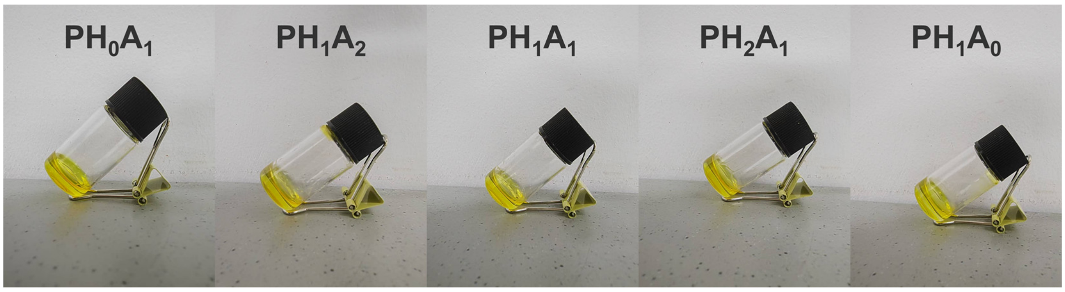

Figure S1. Images of PH_0A_1, PH_1A_2, PH_1A_1, PH_2A_1, and PH_1A_0 after ionogel synthesis.

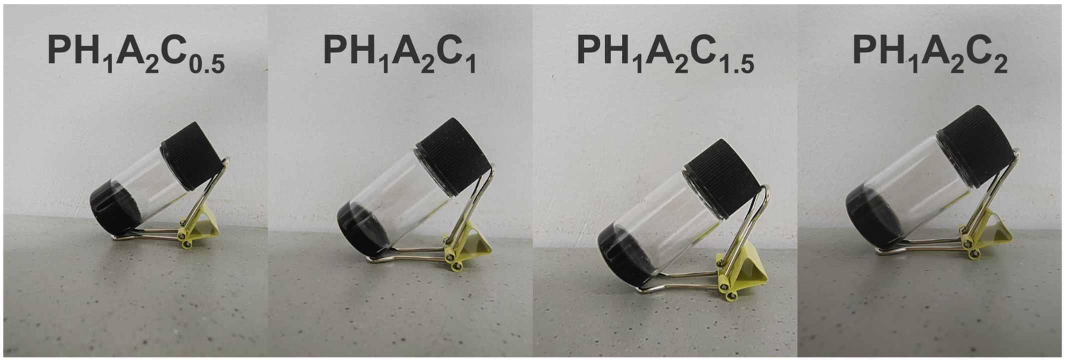

Figure S2. Images of $PH_1A_2C_{0.5}$, $PH_1A_2C_1$, $PH_1A_2C_{1.5}$, and $PH_1A_2C_2$ after ionogel synthesis.

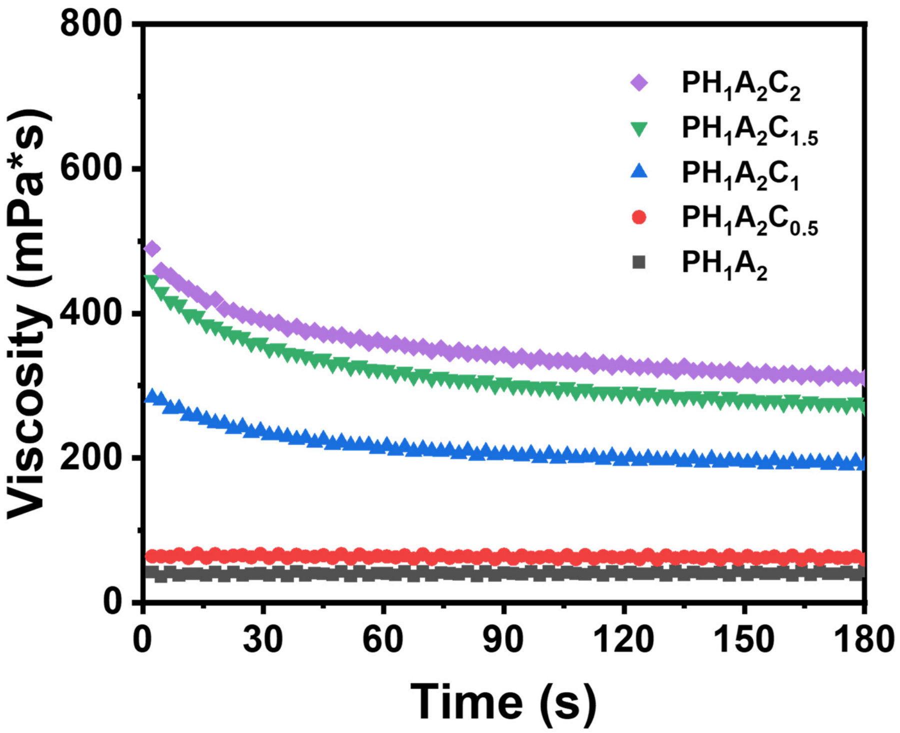

Figure S3. The viscosity of $PH_1A_2C_{0.5}$, $PH_1A_2C_1$, $PH_1A_2C_{1.5}$, and $PH_1A_2C_2$ ionogels.

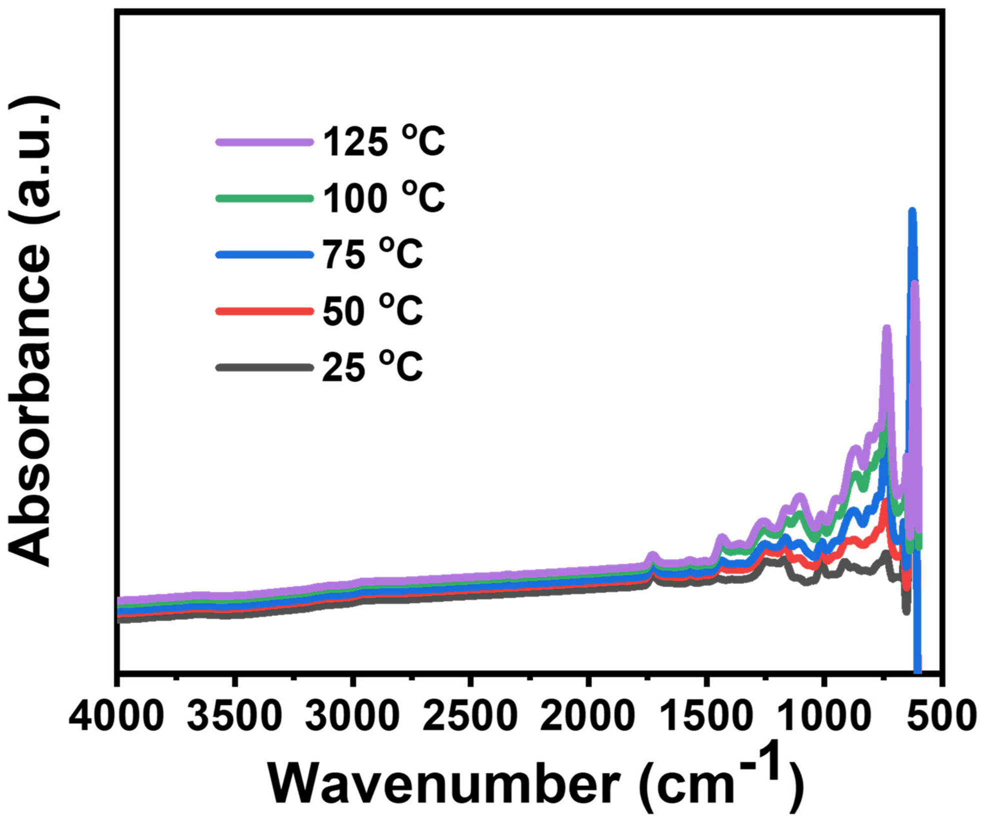

Figure S4. Infrared intensity change curves of P $H_1A_2C_2$ for 4000 cm^{-1} - 400 cm^{-1} at 25 ºC, 50 ºC, 75 ºC, 100 ºC and 125 ºC.

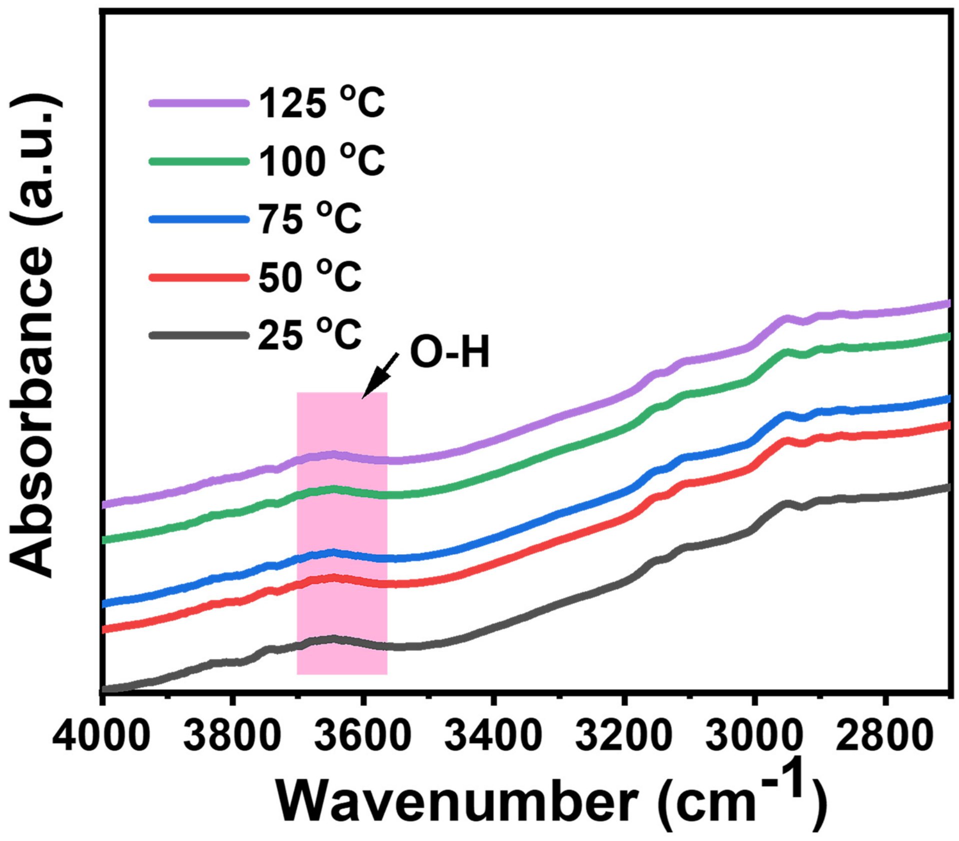

Figure S5. Infrared intensity change curves of P $H_1A_2C_2$ for 4000 cm^{-1} - 2700 cm^{-1} at 25 ºC, 50 ºC, 75 ºC, 100 ºC and 125 ºC.

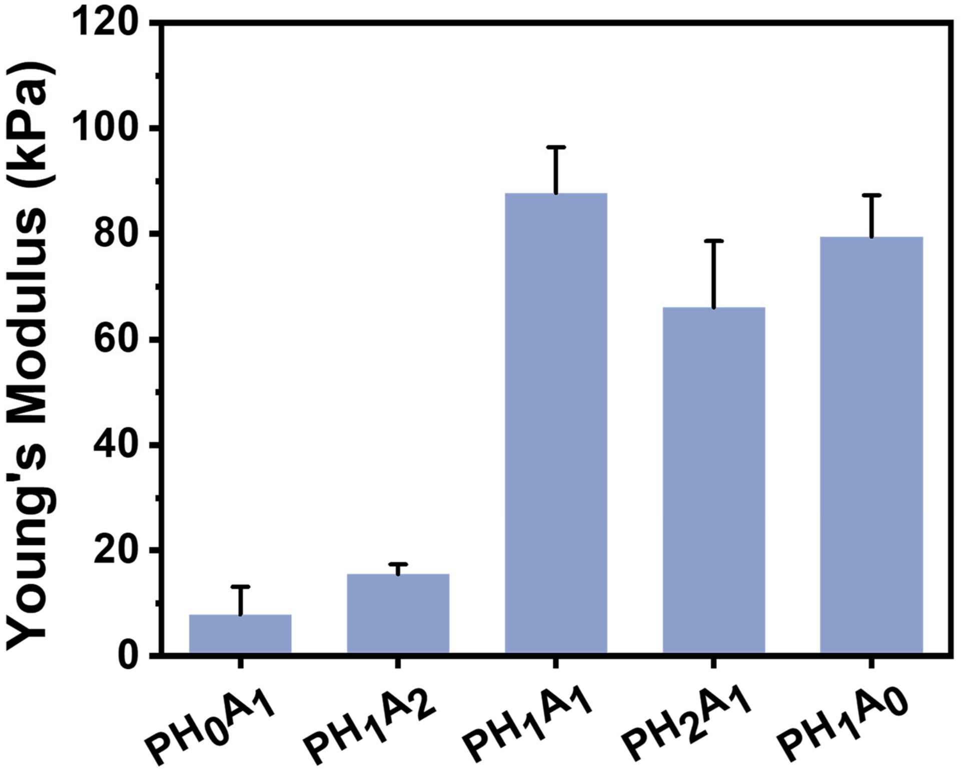

Figure S6. Young's modulus of PH_0A_1, PH_1A_2, PH_1A_1, PH_2A_1, and PH_1A_0 ionogels.

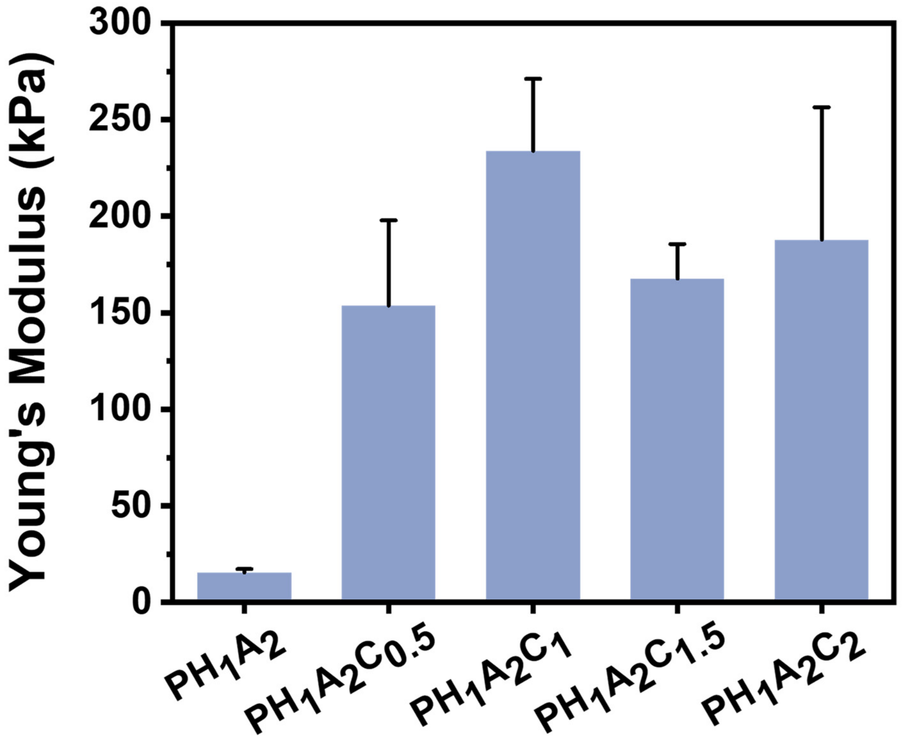

Figure S7. Young's modulus of $PH_1A_2C_{0.5}$, $PH_1A_2C_1$, $PH_1A_2C_{1.5}$, and $PH_1A_2C_2$ ionogels.

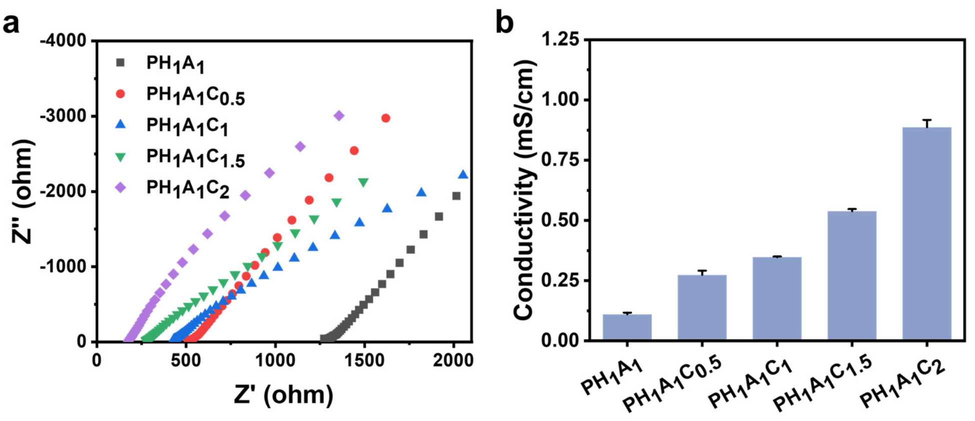

Figure S8. Electrical application characteristics for PHA and PHAC ionogels. a) Nyquist plots of PH_1A_1, $PH_1A_1C_{0.5}$, $PH_1A_1C_1$, $PH_1A_1C_{1.5}$, and $PH_1A_1C_2$ measured by the EIS impedance method. b) Conductivity of PH_1A_1, $PH_1A_1C_{0.5}$, $PH_1A_1C_1$, $PH_1A_1C_{1.5}$, and $PH_1A_1C_2$ calculated from Nyquist plots.

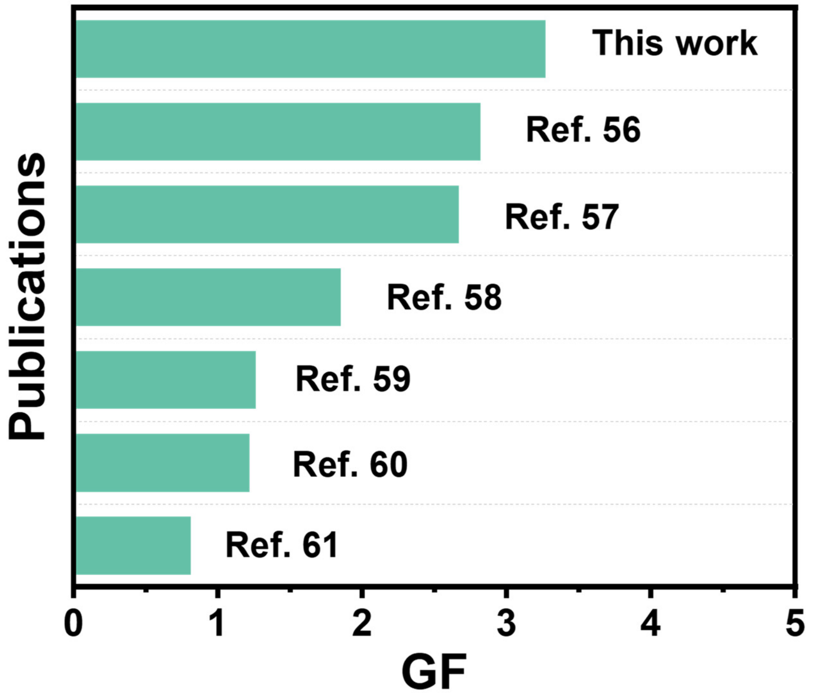

Figure S9. Comparison of GF with previously reported ionogel sensors.

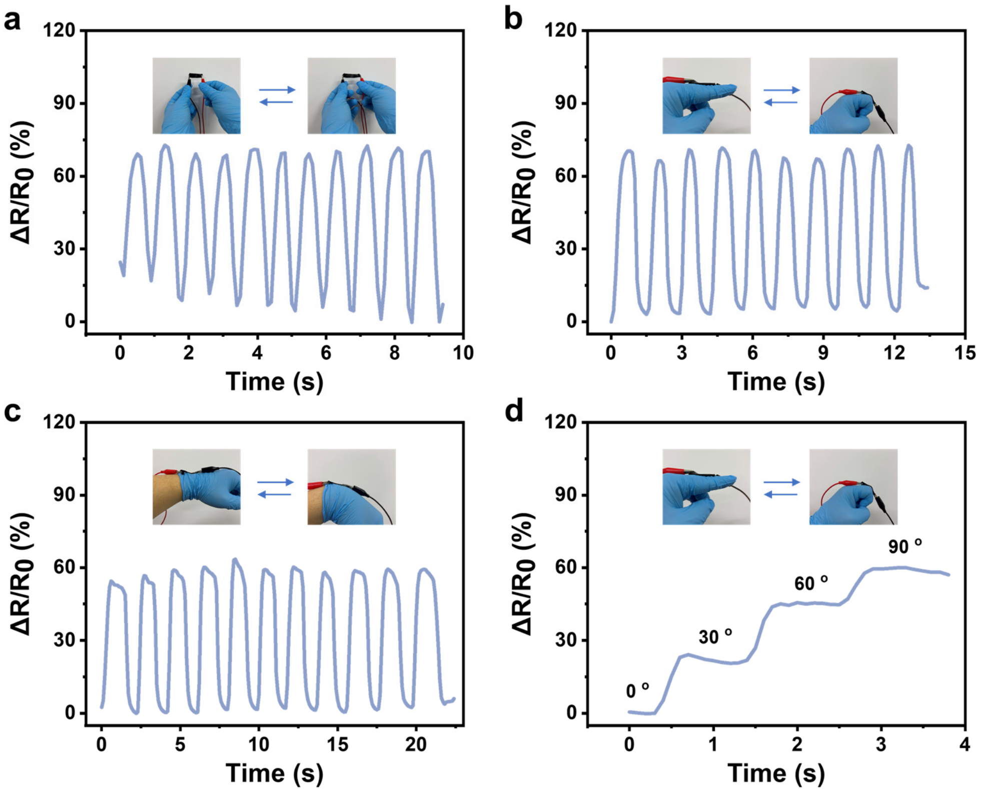

Figure S10. Performance of the ionogel sensors. a) Electrical response of the ionogel sensor when stretched. b) Electrical response of the ionogel sensor when adhered to the finger. c) Electrical response of the ionogel sensor when adhered to the wrist. d) Ionogel sensor producing graded electrical signals depending on the degree of finger bending.

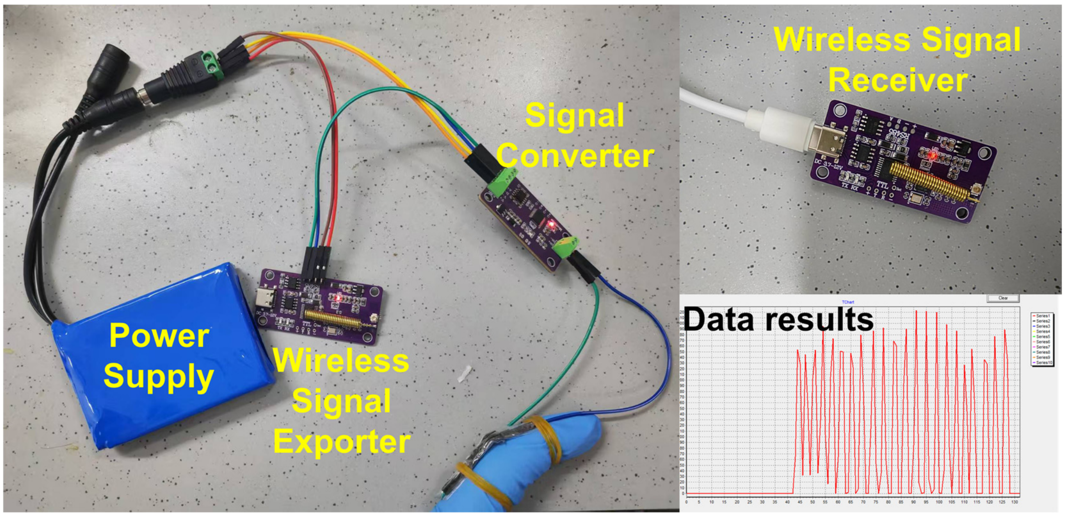

Figure S11. Wireless sensor kit assembled from $PH_1A_2C_2$ ionogel.

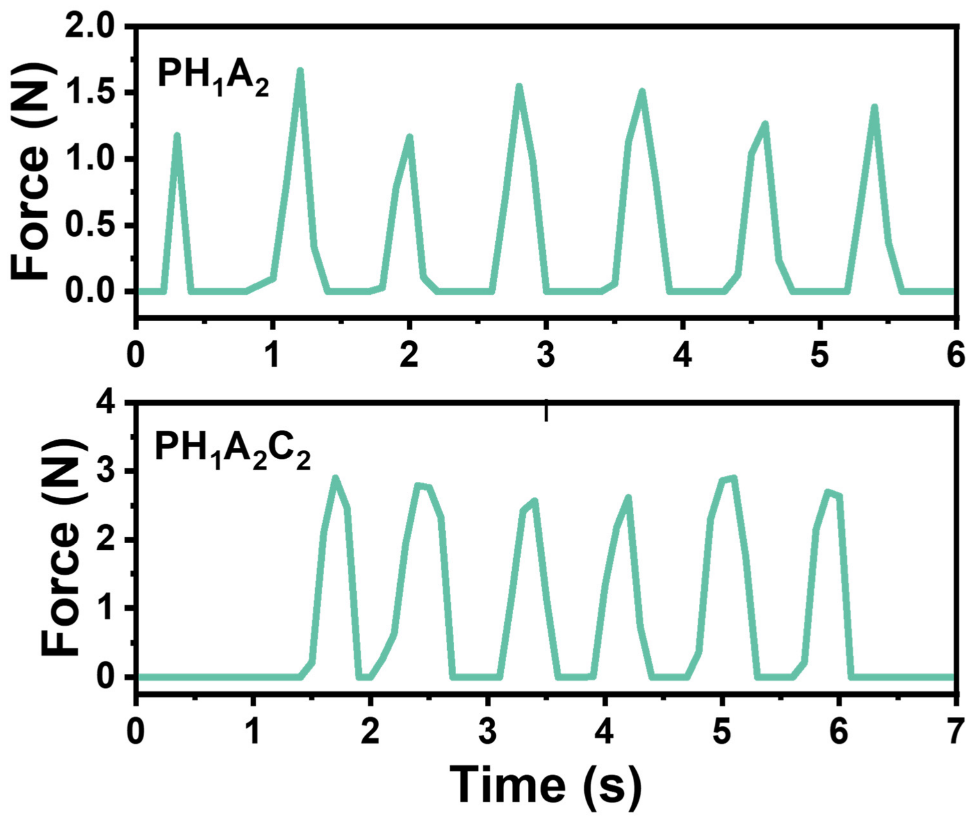

Figure S12. Comparison of the sensing signals of $PH_1A_2C_2$ ionogel and PH_1A_2 ionogel for small finger bends.

Table S1. Formula dosage table of PHA ionogels.

	AA (g)	HEA (g)	EMIES (g)	1173 (g)
PH_0A_1	0.4574	0	1.0000	0.0208
PH_1A_2	0.3050	0.2457	1.0000	0.0208
PH_1A_1	0.2287	0.3686	1.0000	0.0208
PH_2A_1	0.1525	0.4914	1.0000	0.0208
PH_1A_0	0	0.7371	1.0000	0.0208

Table S2. Formula dosage table of PHAC ionogels.

	AA (g)	HEA (g)	EMIES (g)	CNTs (g)	1173 (g)
$PH_1A_2C_{0.5}$	0.3050	0.2457	1.0000	0.0078	0.0208
PH_1A_2 C_1	0.3050	0.2457	1.0000	0.0155	0.0208
PH_1A_2 $C_{1.5}$	0.3050	0.2457	1.0000	0.0233	0.0208
PH_1A_2 C_2	0.3050	0.2457	1.0000	0.0310	0.0208

Table S3. EDS elemental analysis total content of $PH_1A_2C_2$ ionogel.

Elemental	Signal Type	Wt%	Wt% Sigma	At%
C	EDS	49.32	0.95	56.46
N	EDS	10.46	1.32	10.27
O	EDS	37.19	0.84	31.97
S	EDS	3.02	0.11	1.30
Overall amount		100.00		100.00

Table S4. Final results of the multiplication of the signs of each cross-peak in 2DCOS synchronous and asynchronous spectra of the ionogel (Fig. 3d, 3e).

1696	+	+	
1729	-		
1754			
	1754	1729	1696

2DCOS includes two types of correlation maps for both synchronous and asynchronous spectra. Auto peaks appear only on the diagonal of the synchronous spectrum, while cross peaks may appear in both synchronous and asynchronous spectra. The rule for determining the sequence can be summarized as Noda's rule, i.e., if the sign product of the cross peaks (ν_1, ν_2, assuming $\nu_1 > \nu_2$) in the synchronous and asynchronous spectra is positive, the change at ν_1 occurs before ν_2, and vice versa[1-2]. Thus, the order of C=O-related species on heating is determined as (→ indicates prior or earlier): 1729 cm^{-1} → 1754 cm^{-1} → 1696 cm^{-1}.

References

1. Sun, S.-t.; Wu, P.-y., Spectral insights into microdynamics of thermoresponsive polymers from the perspective of two-dimensional correlation spectroscopy. *Chin. J. Polym. Sci.* **2017,** *35* (6), 700-712.
2. Zhang, W.; Wu, B.; Sun, S.; Wu, P., Skin-like mechanoresponsive self-healing ionic elastomer from supramolecular zwitterionic network. *Nature Communications* **2021,** *12* (1), 4082.
